# The Plant Ionome Revisited by the Nutrient Balance Concept

**DOI:** 10.3389/fpls.2013.00039

**Published:** 2013-03-22

**Authors:** Serge-Étienne Parent, Léon Etienne Parent, Juan José Egozcue, Danilo-Eduardo Rozane, Amanda Hernandes, Line Lapointe, Valérie Hébert-Gentile, Kristine Naess, Sébastien Marchand, Jean Lafond, Dirceu Mattos, Philip Barlow, William Natale

**Affiliations:** ^1^Équipe de Recherche en Sols Agricoles et Miniers, Department of Soils and Agrifood Engineering, Université LavalQuébec, QC, Canada; ^2^Department of Applied Mathematics III, Universitat Politècnica de CatalunyaBarcelona, Spain; ^3^Departamento de Agronomia, Universidade Estadual Paulista, Campus de RegistroSão Paulo, Brasil; ^4^Departamento de Solos e Adubos, Universidade Estadual PaulistaJaboticabal, São Paulo, Brasil; ^5^Centre d’Étude de la Forêt, Department of Biology, Université LavalQuébec, QC, Canada; ^6^Centre de Recherches Les BuissonsPointe-aux-Outardes, QC, Canada; ^7^Agriculture and Agri-Food CanadaNormandin, QC, Canada; ^8^Centro de Citricultura Sylvio Moreira (IAC)Cordeirópolis, Säo Paulo, Brazil; ^9^Bio Soil and Crop LtdTauranga, New Zealand

**Keywords:** compositional data analysis, ionome classification, nutrient interactions, numerical biases, isometric log-ratio, plant nutrition

## Abstract

Tissue analysis is commonly used in ecology and agronomy to portray plant nutrient signatures. Nutrient concentration data, or ionomes, belong to the compositional data class, i.e., multivariate data that are proportions of some whole, hence carrying important numerical properties. Statistics computed across raw or ordinary log-transformed nutrient data are intrinsically biased, hence possibly leading to wrong inferences. Our objective was to present a sound and robust approach based on a novel nutrient balance concept to classify plant ionomes. We analyzed leaf N, P, K, Ca, and Mg of two wild and six domesticated fruit species from Canada, Brazil, and New Zealand sampled during reproductive stages. Nutrient concentrations were (1) analyzed without transformation, (2) ordinary log-transformed as commonly but incorrectly applied in practice, (3) additive log-ratio (alr) transformed as surrogate to stoichiometric rules, and (4) converted to isometric log-ratios (ilr) arranged as sound nutrient balance variables. Raw concentration and ordinary log transformation both led to biased multivariate analysis due to redundancy between interacting nutrients. The alr- and ilr-transformed data provided unbiased discriminant analyses of plant ionomes, where wild and domesticated species formed distinct groups and the ionomes of species and cultivars were differentiated without numerical bias. The ilr nutrient balance concept is preferable to alr, because the ilr technique projects the most important interactions between nutrients into a convenient Euclidean space. This novel numerical approach allows rectifying historical biases and supervising phenotypic plasticity in plant nutrition studies.

## Introduction

Salt et al. (2008) defined the ionome as the mineral nutrient and trace element composition of an organism that represents the inorganic component of cellular and organismal systems. The need for linking plant ionomes – often referred as plant nutrient signatures (Willby et al., 2001) or profiles (Tennakoon et al., 2011) – with genetics (Conn and Gilliham, 2010) and adaptation to environmental factors (Chapin, 1989) elevated the study of mineral nutrition of plants as central topic in ecology (Aerts and Chapin, 2000), agronomy (Bergmann, 1988), and genetics (White and Brown, 2010).

The plant ionome is a vector of tissue analytical data generally constrained to the dry or fresh matter content. To facilitate the analysis of complex interacting systems such as the concentration vector of plant ionomes, it is often assumed, under the *ceteris paribus* assumption, that all factors but the ones being varied are equal (Giampietro, 2004). Such assumption denies the principle that components of a whole are inherently related to each other, because changing a proportion inherently affects at least another proportion. In fact, ionome data belong to the class of compositional data, i.e., strictly positive data constrained to some whole, that convey only relative information (Aitchison, 1986). Compositional data are intrinsically multivariate: each part cannot be interpreted without being related to the others (Tolosana-Delgado and van den Boogart, 2011). Indeed, statistics computed across compositional data such as nutrient concentrations are inherently biased due to redundancy, scale-dependency, and non-normal distribution (Bacon-Shone, 2011). Compositional data analysis provides unbiased numerical solutions to analyze plant ionomes as self-interactive systems.

Plant growth and development depend on a balanced supply of essential elements and this equilibrium is maintained by homeostatic mechanisms (Williams and Salt, 2009). Dual ratios (Walworth and Sumner, 1988) and stoichiometric rules (Ingestad, 1987; Körner, 2011) have been proposed to reflect nutrient interactions controlling carbon uptake. Agronomists thus developed a large spectrum of dual (e.g., N/P) and amalgamated (e.g., K/[Ca + Mg]) ratios for diagnostic purposes (Bergmann, 1988). However, one can generate *D*(*D*−1)/2 dual ratios and *D*(*D*−1)^2^/2 amalgamated ratios from a *D*-part ionome, that actually carries *D*−1 degrees of freedom (Aitchison and Greenacre, 2002; Egozcue and Pawlowsky-Glahn, 2005). For example, an ionome including 10 elements generates up to 45 dual nutrient ratios such as the K/Ca ratio and up to 405 amalgamated dual ratios such as the K/(Ca + Mg) ratio, but only nine variables are linearly independent. Researchers realized the great difficulty of interpreting myriads of ratios and proposed integrative empirical models such as the “Diagnosis and Recommendation Integrated System” (DRIS) (Beaufils, 1973). However, DRIS is noisy (Parent et al., 2012a). Although principal component analysis (PCA) also provided a dimension reduction method for nutrient data (Baxter et al., 2008), PCA does not tackle the numerical biases inherent to compositional data (Aitchison, 1986).

Unfortunately, most researchers still use at fault raw concentration data (Lahner et al., 2003; Conn and Gilliham, 2010; White and Brown, 2010), their ordinary log-transformation (Han et al., 2011), or dual ratio expressions when conducting multivariate analyses of ionomes. But fortunately, compositional data analysts have developed log-ratio transformations that generate scale-invariant variables, avoid redundancy, and are free to range in real space (Aitchison, 1986; Egozcue et al., 2003).

Parent and Dafir (1992) were, to our knowledge, the first to correct numerical biases in DRIS using the row-centered log-ratio (*clr*) transformation proposed by Aitchison (1986). The *clr* is computed as ln(*x_i_*/*g*(*x*)), where *x_i_* is the *i*th component (*i* ∈ 1 to *D*) and *g*(*x*) is the geometric mean of the compositional vector. However, matrix singularity occurs in the multivariate analysis, because the *D clr* values add up to zero. As a result, one *clr* value must be removed. Other log-ratio transformations can compress a D-part composition into D–1 variables without losing information, hence avoiding singularity problems.

Aitchison (1986) proposed using the additive log-ratio (*alr*) computed as ln(*x_j_*/*x_A_*) where *x_j_* is the *j*th component (*j* ∈ 1 to *D* except *A*) and *x_A_*, the common denominator of the compositional vector. The *alr* transformation can reflect the stoichiometric rules used in plant physiology and nutrient management (Ingestad, 1987). However, *alr* variables are at an angle of 60° between them and are thus geometrically difficult to handle (Egozcue and Pawlowsky-Glahn, 2006).

A dual ratio between two nutrients is a dual balance, hence removing one variable while keeping all the relevant information. An extended balance system can be illustrated by a ternary diagram (Lagatu and Maume, 1934) or by a mobile and its fulcrums built according to an *ad hoc* scheme for several components (Parent et al., 2012a). In compositional data analysis, balances are expressed as log-ratio contrasts between the geometric means of two parts or groups of parts (Egozcue and Pawlowsky-Glahn, 2005). Assigning orthogonal coefficients to contrasts allows computing orthogonal balances as isometric log-ratio (*ilr*) in the Euclidean space (Egozcue et al., 2003). The *ilr* technique was found to be the most appropriate to describe natural patterns in geochemistry (Buccianti, 2011), plant nutrition (Parent, 2011; Parent et al., 2012c), environmental sciences (Filzmoser et al., 2009a), soil physics (Parent et al., 2012b), chemistry and biochemistry (Parent et al., 2012a), and other disciplines (Pawlowsky-Glahn and Buccianti, 2011).

Our objective is to present an unbiased balance concept to the plant nutrition community using data sets of fruit species, to illustrate and provide a robust perspective to solve the important problem of data representation when conducting multivariate analysis of ionomes. We hypothesize that ionomes of wild (low phenotypic plasticity) and domesticated (high phenotypic plasticity) species differ markedly from each other due to natural adaptation or human selection pressure (Chapin, 1989). We expand the nutrient balance concept to species and cultivars.

## Theory of Compositional Data Analysis

### Sample space

The sample space (e.g., the space of compositional data of plant ionomes reported on dry mass basis) is defined by *S^D^*, a positive vector of *D* components adding up to a constant κ, such as 1 (fractions of some whole), 100% (e.g., N-P-K ternary diagram representing an ionome subcomposition), 1000 g kg^−1^ (e.g., sum of nutrient concentrations and of the filling value in an ionome), etc. The closure operator *C* computes the constant sum assignment as follows (Egozcue and Pawlowsky-Glahn, 2006):

(1) $$\begin{array}{llll} S^{D} & =C\;\left(c_{1},\;c_{2},\;\ldots ,\;c_{D}\right) & & \\ & =\left[\frac{c_{1}K}{\sum_{i=1}^{D}c_{i}},\;\frac{c_{2}K}{\sum_{i=1}^{D}c_{i}},\;\ldots ,\;\frac{c_{D}K}{\sum_{i=1}^{D}c_{i}}\right] \end{array}$$

where κ is the unit or scale of measurement and *c_i_* is the *i*th part of a composition containing *D* parts. The ionome comprises analytical results as well as, optionally, undetermined concentrations of other elements summarized by the filling value. The filling value is computed by difference between the unit or scale of measurement and the sum of analytical results. The sample space can be subdivided into non-overlapping subspaces made of two (dual ratios), three (ternary diagram), or more interacting components where each subspace can be interpreted independently and coherently.

### Numerical biases

The redundancy and scale-dependency inherent to compositional data generate spurious correlations (Pearson, 1897; Tanner, 1949; Chayes, 1960) that distort their multivariate analysis (Aitchison, 1986). The multivariate analysis of concentration values or their ordinary log transformation may thus lead to biased and even meaningless results (Filzmoser et al., 2009b). These biases can be avoided using compositional data analysis techniques (Egozcue and Pawlowsky-Glahn, 2006; Mateu-Figueras et al., 2011).

Redundancy can be avoided by (1) sacrificing a component for use as common denominator (*alr* transformation) (Aitchison, 1986) or (2) using the principle of contrasts orthogonality whereby the orthogonally arranged balances acquire linear independence (Rodgers et al., 1984) using *ilr* transformation (Egozcue et al., 2003). The isometry of *ilr* variables means that the geometry is Euclidean, which is the very basic geometry in multivariate analysis (Egozcue and Pawlowsky-Glahn, 2006).

Scale invariance assures that data have the same covariance structure no matter the base across which they are scaled, e.g., across wet, dry, organic, mineral or macronutrient basis. Scale invariance is required to provide a coherent interpretation of multivariate analyses of compositional data (Aitchison, 1986; Egozcue and Pawlowsky-Glahn, 2005).

Non-normal distribution inherent to compositional data is improved by projecting the constrained space of raw compositional data into a real space of log-ratios. Because log-ratios can take any value in the domain ±∞, *alrs* and *ilrs* can be mapped in real space, as required under the normality assumption (Egozcue and Pawlowsky-Glahn, 2006). By comparison, confidence intervals that may reach values <0 or beyond 100% under the normality assumption have no physical meaning (Weltje, 2002). The log-ratio transformation improves normal distribution compared to raw concentrations or their ordinary log-transformation (Filzmoser et al., 2009a). All in all, the *ilr* transformation is recommended for conducting multivariate analyses of compositional data (Filzmoser and Hron, 2011).

### The log-ratio transformations

#### The *alr* transformation

Log transforming the P/N, K/N, Ca/N, and Mg/N ratios elaborated by Ingestad (1987) to monitor the plant nutrition of tree seedlings yield *D*−1 *alr* variables. The number of degrees of freedom is reduced by using one component as common denominator. The choice of the common denominator has no influence on multivariate analysis (Aitchison, 1986). In the Ingestad (1987) model, the common basis is *N* concentration. The *j*th *alr* is computed as follows:

(2) $$alr_{j}=\ln\frac{c_{j}}{N}$$

where *c_j_* is the *j*th nutrient excluding N. If a tissue contains 2.50% N and 0.15% P, the Redfield ratio (Güsewell, 2004) is 16.7 and the corresponding *alr* [P/N] value is ln(0.15/2.50) = −2.81. However, the *alrs* are oblique to each other and difficult to rectify (Egozcue and Pawlowsky-Glahn, 2005).

#### The *ilr* transformation

The *ilr* transformation has the advantage over the *alr* to be geometrically suited to conduct multivariate analysis (Filzmoser et al., 2009a). Another advantage of *ilrs* is a special device of balances or linearly independent ratios among nutrients called sequential binary partition (SBP). The SBP describes the *D*−1 orthogonal (geometrically independent) balances between parts and groups of parts. The SBP is a (*D*−1) × *D* matrix, in which parts labeled “+1” (group numerator) are balanced with parts labeled “−1” (group denominator). A part labeled “0” is excluded from the balance between parts. The composition is partitioned sequentially into contrasts at every hierarchically ordered row until the (+1) and (−1) groups each contain a single part.

To establish contrast orthogonality, it is necessary to imbed subcompositions into larger ones and assign orthogonal coefficients to each log-ratio contrast (Egozcue et al., 2003). The *ilr* is computed as follows (Egozcue and Pawlowsky-Glahn, 2005):

(3) $$ilr_{j}=\sqrt{\frac{r_{j}s_{j}}{r_{j}+s_{j}}}\ln\frac{g\left(c_{j}^{+}\right)}{g\left(c_{j}^{-}\right)}$$

where, in the *j*th row of the SBP, *ilr_j_* is the *j*th isometric log-ratio; $\sqrt{r_{j}s_{j}/r_{j}+s_{j}}$ is the orthogonal coefficient of the *j*th balance (or log contrast) designed in the SBP; *r_j_* and *s_j_* represent the number of parts in the +1 and −1 groups of the *j*th balance, respectively; $g(c_{j}^{+})$ is the geometric mean of components in the +1 group and $g(c_{j}^{-})$ is the geometric mean of components in the −1 group. The partition between two components or groups of components is presented as [*S^r^* | *S^s^*]. If a tissue contains 2.50% N and 0.15% P, the Redfield ratio (Güsewell, 2004) is 16.7 and the *ilr* value is $[N|P]=\sqrt{\frac{1}{2}}\ln\;(16.7)=1.99.$

### Nutrient balances

**Figure 1:**
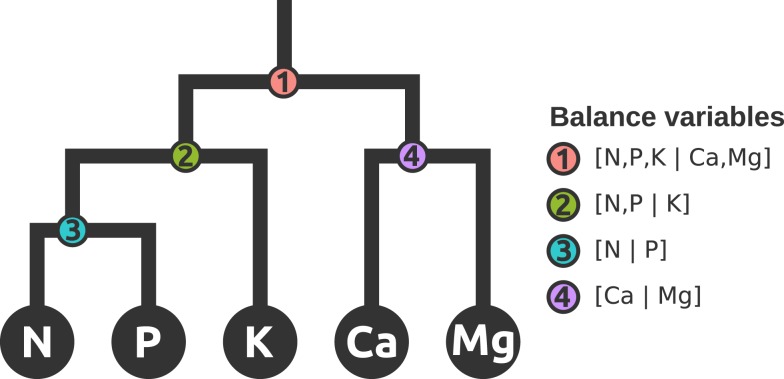
**Mobile-and-fulcrums at mass equilibration point illustrates four hierarchically nested balances that represent a subcomposition or subspace of nutrients in the ionome**.

Nutrient balances are robustly amenable to myriads of statistical techniques analysis: balances variables are non-redundant and scale-invariant, are mapped in a real space and respect the *D*−1 degrees of freedom of a compositional vector. The SBP allows the analyst to define orthogonal axes in order to focus upon interpretable balances. In order to compare the ionomes of plant species, a subcomposition of the compositional vector was defined as *S*^5^ = *C* (N, P, K, Ca, Mg). The SBP in Table 1 formalizes balance dendrograms such as the one presented in Figure 1. In a more intuitive but similar approach, nutrient balance could be illustrated by a mobile-and-fulcrums design where nutrient concentrations in weighing pans are equilibrated according to *ad hoc* nutrient balances.

**Table 1:** **Sequential binary partition (SBP) elaborated to compute balances between groups of nutrients as isometric log-ratios (*ilr*)**.

ilr	SBP contrasts						Balance designation	*r*^†^	*s*^†^	Ilr computation^‡^
	N	P	K	Ca	Mg	*F_v_*	
ilr_1_	+1	+1	+1	−1	−1	0	[N, P, K | Ca,Mg]	3	2	$\sqrt{\frac{3\times 2}{3+2}}\ln\frac{g\left(c_{N}c_{P}c_{K}\right)}{g\left(c_{ca}c_{Mg}\right)}$
ilr_2_	+1	+1	−1	0	0	0	[N, P | K]	2	1	$\sqrt{\frac{1\times 2}{1+2}}\text{ ln }\frac{g(c_{N}c_{P})}{g(c_{K})}$
ilr_3_	+1	−1	0	0	0	0	[N | P]	1	1	$\sqrt{\frac{1\times 1}{1+1}}\text{ ln }\frac{g(c_{N})}{g(c_{P})}$
ilr_4_	0	0	0	+1	−1	0	[Ca | Mg]	1	1	$\sqrt{\frac{1\times 1}{1+1}}\text{ ln }\frac{g(c_{ca})}{g(c_{Mg})}$
Optional	1	1	1	1	1	−1	[N, P, K, Ca, Mg | *F*_υ_]	5	1	$\sqrt{\frac{5\times 1}{5+1}}\ln\frac{g(c_{N}\;c_{P}\;c_{K}\;c_{ca}\;c_{Mg})}{g(c_{F\upsilon })}$

*An optional balance is a computed as a contrast between the geometric mean of nutrient analyses and the filling value, computed by difference*.

*^†^*r* and *s* are counts of +1 and −1 elements in the balance, respectively, ^‡^*g* is geometric mean*.

There are indeed four orthogonal balances in *S*^5^ (Figure 1). Our SBP initiator was [N, P, K | Ca, Mg] to reflect sequentially the relationships between N, P, and K (Lagatu and Maume, 1934; Wilkinson et al., 2000) in agroecosystems, the Ca and Mg composition that reflects geographical position and soil mineralogy (Walworth and Sumner, 1987), and the Redfield ratio that reflects the balance between two fundamental life processes, protein, and r-RNA synthesis (Loladze and Elser, 2011).

### The Aitchison distance

The Aitchison distance (𝒜) between two *D*-part compositions is computed as a Euclidean distance across selected *ilr* coordinates as follows (Egozcue and Pawlowsky-Glahn, 2006):

(4) $$A=\sqrt{\overset{D-1}{\underset{j=1}{\sum }}{\left(A_{ilr_{j}}-B_{ilr_{j}}\right)}^{2}}$$

where $A_{ilr_{j}}$ and $B_{ilr_{j}}$ are the *j*th *ilr* coordinate of the composition of two rows *A* and *B*. If one of the two rows is a null vector, 𝒜 is called the Aitchison norm.

If Euclidean geometry is not valid, arithmetic mean is likely to be a poor estimate of data center (Filzmoser et al., 2009a). Even after ordinary log transforming compositional data, the squared Euclidean distance (ε^2^) between ordinary log-transformed compositions *x* and *y*, i.e., between ln(*x*) and ln(*y*), is always equal to or greater than the squared 𝒜 distance between the *ilrs* of compositions *x* and *y* (Eq. 4) as driven by the number of components and their geometric means *g*(*x*) and *g*(*y*), as follows (Lovell et al., 2011):

(5) $$\varepsilon^{2}\left[\ln\left(x\right),\;\ln\left(y\right)\right]=A^{2}+D\times {\left(\ln\left(\frac{g\left(x\right)}{g\left(y\right)}\right)\right)}^{2}\geq A^{2}$$

Numerical biases can be measured as a positive shift from 𝒜 to ε. Note that ε^2^ = 𝒜^2^ only when *g*(*x*) = *g*(*y*). As a result, computing univariate or multivariate distances across raw or ordinary log-transformed concentration data is geometrically irrelevant (Aitchison, 1986).

## Materials and Methods

### Datasets

The selected fruit species were either wild (lowbush blueberry and cloudberry) or domesticated to achieve high productivity (other species). Nutrient data were collected for kiwifruit [*Actinidia deliciosa* (A Chev) C F Liang et A R Ferguson var deliciosa] grown in the North Island of New Zealand, guava (*Psidium guajava*), orange (*Citrus sinensis*), and mango (*Mangifera indica*) grown in the state of São Paulo, Brazil, and apple (*Malus domestica* Borkh.), cranberry (*Vaccinium macrocarpon* Ait.), lowbush blueberry (*Vaccinium angustifolium* Ait.), and cloudberry (*Rubus chamaemorus* L.) from the province of Quebec, Canada.

The number of observations was comparable or less compared to other studies on mango (*n* = 525 collected in a single year: Schaffer et al., 1988), hazelnut (*n* = 624 collected over 16 year: Alkoshab et al., 1988), sweet cherry (*n* = 475 collected over 3 year: Davee et al., 1986), and orange (*n* = 3161 collected over 21 year: Beverly et al., 1984). Leaf samples from 4 to 32 plants were composited in each plot or orchard area to minimize between-plant variability, compared to one tree in other studies.

Marschner (1995) claimed that physiological age of a plant or plant part is, next to mineral nutrient supply, the most important factor affecting plant nutrient concentration. Across-season samplings (e.g., Han et al., 2011) thus influence nutrient concentrations (Bould, 1968) as well as ratios (Güsewell, 2004). The developmental stage for sampling occurs during phases of minimum or indeterminate nutrient changes in the fully developed leaves (Bould, 1968). Therefore sample collection must be completed within a short period of time to minimize seasonal variability (Willby et al., 2001). Foliar samplings of fruit-bearing shoots were performed during the reproductive stages either at full bloom (guava, mango), after flowering (kiwifruit), during fruit development (orange, apple), during fruit maturation (cranberry, blueberry), or from fruit set to maturity (cloudberry).

Two to three of the youngest fully expanded leaves were collected from 32 vines (excluding young vines and sick leaves) on the second lateral cane within 0 to 4 weeks in 908 commercial “Hort 16a Gold” and “Hayward” kiwifruit orchards in the North Island of New Zealand during the 2002–2010 period. Fruit yield averaged 31800 kg ha^−1^. The climate is subtropical humid, and soils are Andisols of volcanic origin.

Guava, mango, and orange yields and nutrient data were collected in the state of São Paulo, Brazil. Thirty pairs of leaves around each of 25 trees were composited (Quaggio et al., 1997). A survey was conducted on 137 irrigated “Paluma” guava orchards (three cycles per 2 years) during the 2009–2010 and 2010–2011 production cycles. Fruit yield averaged 56155 kg ha^−1^. From 2009 to 2011, leaf data were collected in 95 mango orchards where varieties “Espada,” “Palmer,” and “Tommy” were grown. Fruit yield averaged 15700 kg ha^−1^. Foliar samples were collected between 1978 and 2005 in 104 orange orchards producing the varieties “Valencia,” “Hamlin,” “Pêra,” and “Natal.” Fruit yield averaged 49300 kg ha^−1^. The climate is subtropical humid, and the soils are Oxisols and Ultisols of basaltic origin.

Apple data of the “Morspur McIntosh” variety were obtained from an N, P, K, Ca and Mg fertilizer trial (576 observations) established in southwestern Quebec, Canada (Parent and Granger, 1989). Ten to 30 leaf samples were collected in the middle of the annual growth. Fruit yield averaged 33600 kg ha^−1^. Climate is temperate humid continental and soils are Spodosols of morainic origin.

Cranberry (cv. “Stevens”) yield and nutrient data were collected at five sites in Central Quebec, Canada (Parent and Marchand, 2006) in 2000, 2001, and 2002, for a total of 149 observations. One-hundred leaves from current season stems were sampled randomly in a 1-m^2^ plot. Berry yield averaged 28200 kg ha^−1^. The climate is temperate humid continental, and soils are Spodosols of marine origin.

Yield and nutrient of lowbush blueberry totaling 345 observations were collected from 2001 to 2006 in eight commercial fields in northern Quebec, Canada (Lafond, 2009). Leaf tissues from 25 randomly selected stems were sampled in the 2001, 2003, and 2005 sprout (vegetative) years in 50-m^2^ plots and composited. Berry yield averaged 3600 kg ha^−1^. The climate is cold, and soils are Spodosols developed on deltaic deposits.

In 2009, cloudberry leaves were collected in 86 stands of contrasting productivity along the Lower North Shore of the St. Lawrence River, Quebec, Canada (Hébert-Gentile et al., 2011). Six shoots were randomly selected in 5-m^2^ plots. The median fruit yield was 35 kg ha^−1^. The climate is cold, and soils are Histosols developed on ombrotrophic peat lands covered with sphagnum (wetter areas) or lichens (drier areas).

### Tissue analysis

Tissue P, K, Ca, and Mg levels in the leaves of kiwifruit were determined by plasma emission spectroscopy after microwave digestion (Blackmore et al., 1987). Total *N* was determined by dry combustion using a Leco CNS-2000 analyzer (Leco, St. Joseph, MI, USA). For guava, orange and mango, tissue *N* was determined by micro-Kjeldahl and P, K, Ca, and Mg by ICP-OES after digestion in a mixture of nitric and perchloric acids (Bataglia et al., 1983; Jones and Case, 1990). For apple, total *N* was determined by micro-Kjeldahl and other nutrients colometrically (P) or by AA spectrophotometry (K, Ca, Mg). For cranberry, blueberry, and cloudberry leaves, total *N* was determined by micro-Kjeldahl digestion or by Leco CNS-2000 combustion. Other elements were quantified by colorimetry (P), AA spectrometry, or ICP-OES after digestion in a mixture of perchloric and nitric acids (Jones and Case, 1990).

### Statistical analysis

Statistical computations were conducted in the *R* statistical environment (R Development Core Team, 2011). Compositional data analysis was conducted using the *R* “compositions” package (van den Boogaart et al., 2011). Multivariate outliers were removed for robust multivariate analysis (Filzmoser et al., 2008) using the Mahalanobis distance at a 0.01 level of significance with the *R* “mvoutlier” package (Filzmoser and Gschwandtner, 2011). Data distribution was tested with the Anderson–Darling normality test (Thode, 2002) using the “nortest” package (Gross, 2006). Spurious correlations were reported as Pearson correlation coefficients. Discriminant analysis (DA) was conducted with the *R* “ade4” package (Chessel et al., 2011) to compare the classification of plant nutrient signatures of wild and domesticated species.

## Results

### Distribution, scale-dependency, and spurious correlations

**Figure 2:**
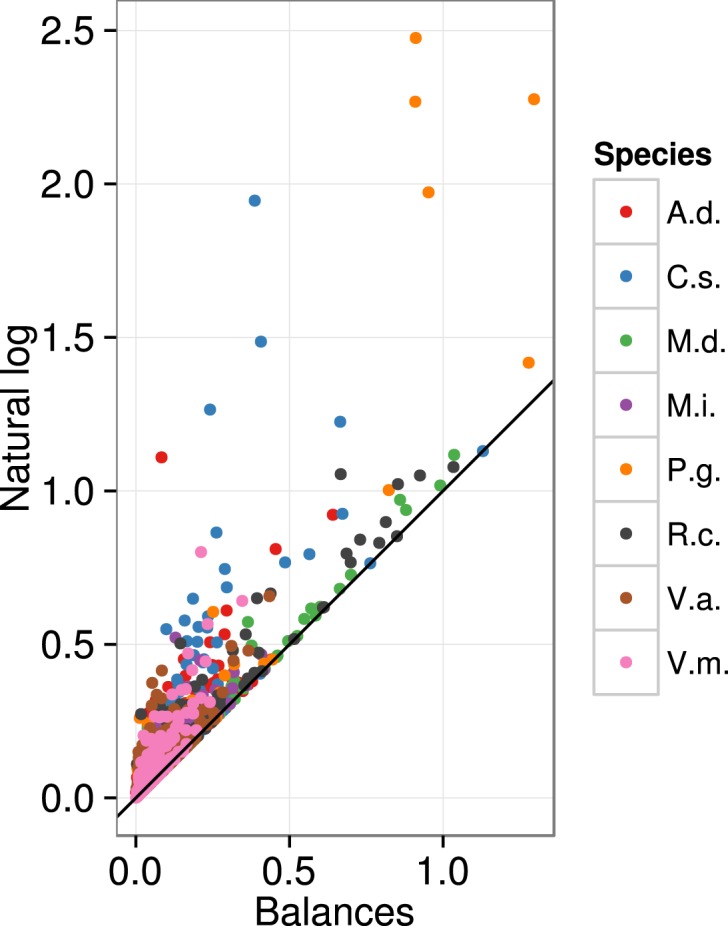
**Numerical biases are illustrated by the inflated Euclidean distance across ln-transformed five nutrient concentrations compared to *ilr* transformation across four balances**. A.d., kiwifruit [*Actmidia deliciosa* (A Chev) C F Liang et A R Ferguson var deliciosa]; C.s., orange (*Citrus sinensis*); M.d., apple (*Malus domestica* Borkh.); M.i., mango (*Mangifera indica*); P.g., guava (*Psidium guajava*); R.c., cloudberry (*Rubus chamaemorus* L.); V.a., lowbush blueberry *(Vaccinium angustifolium* Ait.); V.m., cranberry (*Vaccinium macrocarpon* Ait.).

The Euclidean distance computed across ordinary log-transformed concentration data was higher and more dispersed compared to the Aitchison distance across balances (Figure 2). This discrepancy is a measure of numerical biases in multivariate analysis of compositional data using ordinary log transformations.

**Figure 3:**
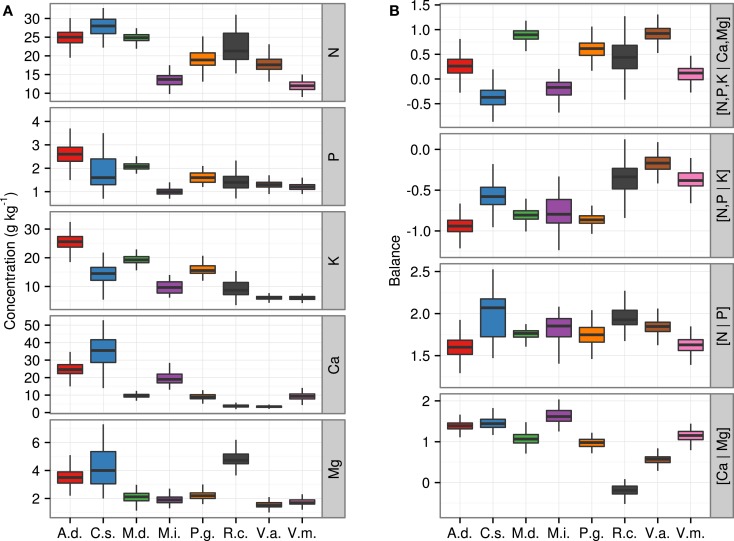
**Boxplots of ionomes of eight fruit plant species (A) across nutrient concentrations and (B) across *ilr* balances**. A.d., kiwifruit [*Actmidia deliciosa* (A Chev) C F Liang et A R Ferguson var deliciosa]; C.s., orange (*Citrus sinensis*); M.d., apple (*Malus domestica* Borkh.); M.i., mango (*Mangifera indica*); P.g., guava (*Psidium guajava*); R.c., cloudberry (*Rubus chamaemorus* L.); V.a., lowbush blueberry *(Vaccinium angustifolium* Ait.); V.m., cranberry (*Vaccinium macrocarpon* Ait.).

Moreover, 70% of the *ilrs* were normally distributed (*p*-value < 0.01). The [N | P] balance was the most frequently diagnosed as non-normally distributed. Most other balances (84%) were normally distributed across species. By comparison, only 33 and 35% of the raw or ordinary log-transformed concentrations values, respectively, were normally distributed. Data distributions of nutrient concentrations and balances (*ilr*) are presented in the form of box plots in Figure 3 for the eight species. The mean of *ilr* often differed between species as shown by non-overlapping ranges.

Besides, correlation coefficients changed in magnitude, sign, or probability level depending on the choice of the scale of nutrient expressions (sum of nutrients vs. dry matter basis) (Table 2). Scale-dependency causes a serious problem of interpretation when statistical analyses are based on the covariance or correlation matrix.

**Table 2:** **Correlation matrices of nutrient data of *Malus domestica* computed across two scales: dry matter content and N-P-K-Ca-Mg**.

Scale	Nutrients	N	P	K	Ca	Mg
Data scaled on dry matter content (common expression)	N	1	0.023	0.068ns	0.232**	0.271**
	P		1	−0.003ns	0.138**	0.220**
	K			1	−2.38**	−0.205**
	Ca				1	0.080ns
	Mg					1
Data scaled on the sum (N + P + K + Ca + Mg)	N	1	−0.029ns	−0.591**	−0.245**	0.293**
	P		1	−0.219**	−0.003ns	0.200**
	K			1	−0.574**	−0.455**
	Ca				1	−0.017ns
	Mg					1

ns, *, **: non-significant and significant at the 0.05 and 0.01 levels, respectively.

### Discriminant analysis

**Figure 4:**
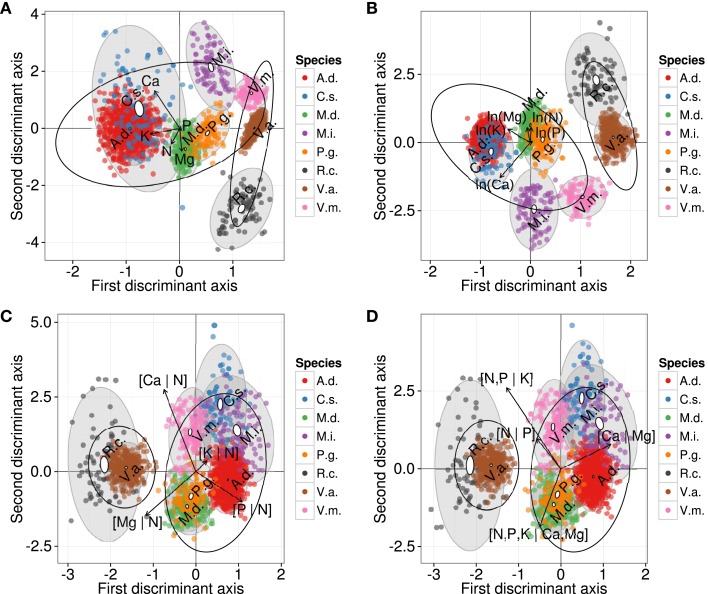
**Discriminant analysis of ionomes by species using (A) raw concentrations, (B) ln-transformed concentration values, (C) additive log-ratios, and (D) isometric log-ratio balances**. Large semitransparent ellipses that enclose swarms of data points represent regions that include 95% of the theoretical distribution of canonical scores for each species. Smaller plain white ellipses represent confidence regions about means of canonical scores at 95% confidence level. Empty ellipses represent data swarms for wild and domesticated species, respectively. A.d., kiwifruit [*Actmidia deliciosa* (A Chev) C F Liang et A R Ferguson var deliciosa]; C.s., orange (*Citrus sinensis*); M.d., apple (*Malus domestica* Borkh.); M.i., mango (*Mangifera indica*); P.g., guava (*Psidium guajava*); R.c., cloudberry (*Rubus chamaemorus* L.); V.a., lowbush blueberry *(Vaccinium angustifolium* Ait.); V.m., cranberry (*Vaccinium macrocarpon* Ait.).

The DAs returned different schemes whether nutrients were expressed as raw concentrations or their ordinary log transformations (Figures 4A,B). Large semitransparent ellipses enclosing swarms of data points represent regions that include 95% of the theoretical distribution of canonical scores for each ionome. The swarms of wild and domesticated species (large ellipses) overlapped using raw concentration data, but were separated using ordinary log transformation of concentrations. The smaller plain white ellipses represent the confidence regions about the mean of canonical scores at the 95% confidence level. Plain white ellipses related to A.d. (kiwifruit) and V.a. (lowbush blueberry) were too small to be visible. Mean nutrient signatures differed significantly between species because the white ellipses did not overlap, indicating plant-specific ionomes. Eigen vectors were similar between raw and ordinary log-transformed data, where K and Ca loaded most on the first axis.

While DAs of the ordinary log, *alr* and *ilr* representations led to a separation between wild and domesticated species, the swarms of species were positioned differently in the Euclidean space (Figures 4C,D). The unbiased *alr*- and *ilr*-based DAs were almost identical. Differences are imputed to different outlier detection results caused by the different geometries of *alr* and *ilr*. Figures 4C,D showed that some ionome distributions (semitransparent gray ellipses) overlapped, while the confidence regions about means (white ellipses) differed significantly between species. In the *alr*-based DA, [Mg | N] and [P | N] loaded the most on the first axis. In the *ilr*-based DA, [N, P | K], related to nutrient management in agroecosystems, and [Ca | Mg], related to geographical position as well as soil liming in agroecosystems, loaded the most on the first axis. Although the way nutrient balances are arranged into *alr* or *ilr* variables produced almost identical DAs, interpretation of results depended on data representation as log ratios and this emphasizes the importance of sound data representations when conducting multivariate analysis of compositional data.

**Figure 5:**
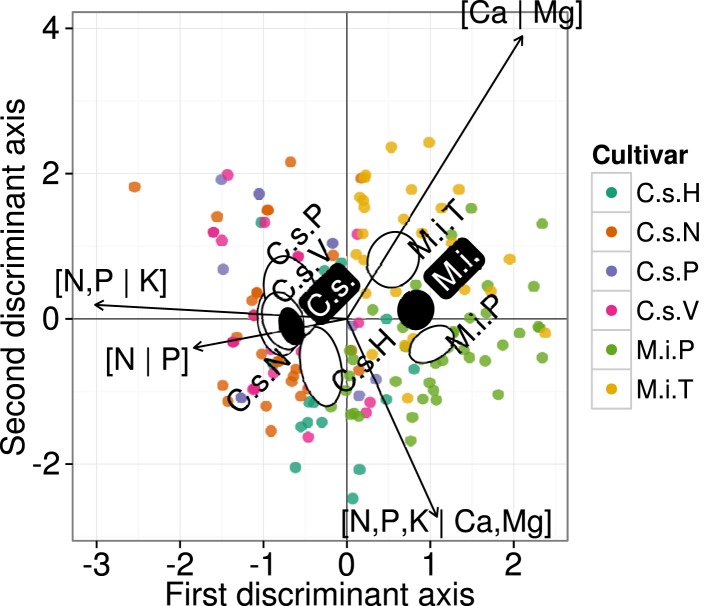
**Discriminant analysis of ionomes by cultivar using isometric log-ratio balances**. Large semitransparent ellipses that enclose swarms of data points represent regions that include 95% of the theoretical distribution of canonical scores for each cultivar. Smaller plain white ellipses represent confidence regions about means of canonical scores at 95% confidence level. Empty ellipses represent data swarms for mango and orange species, respectively. Orange (*Citrus sinensis*): H, Hamlin; N, Natal; P, Pera; V, Valencia. Mango (*Mangifera indica*); P, Palmer; T, Tommy.

On the other hand, the small ellipses of species were at significant distance from each other (*p* < 0.05). In a finer analysis at cultivar level, DA of the ionomes that were averaged across cultivars of orange and mango (Figure 5) indicated significant differences (*p* < 0.05) between means of discriminant scores of orange cv. “Hamlin” and others, and between mango cvs, “Palmer,” and “Tommy,” indicating genotypic differences or phenotypic adjustment of each species to local factors.

## Discussion

### Unbiased analysis of plant ionomes

The DAs performed using unstructured raw or ordinary log-transformed concentration data posed serious interpretation problems in the multivariate analysis of plant ionomes. First, the normality assumption is violated intrinsically by the constrained compositional space. Second, as a result of scale-dependency, the multivariate analysis can differ by simply changing the dry mass basis for another denominator such as the wet mass (Walworth and Sumner, 1988) or the sum of nutrients. Third, one may conclude that Ca and K concentrations are the most discriminant variables, but K concentration is inherently connected to Ca in plant nutrition (Wilkinson et al., 2000). Indeed, K bears redundant information about Ca because K and Ca interact in the plant and are thus inherently correlated to each other: indeed, Ca may decrease as K concentration increases in the confined compositional space as driven by K antagonism or luxury consumption (Marschner, 1995). Compositional data analysis avoids redundancy by relating Ca to K in linearly independent (i.e., orthogonally arranged) log-ratios.

In addition to the numerical advantages of *ilr* discussed above, nutrient balances also reflect nutrient interactions, which are generally neglected in the multivariate analysis of plant ionomes. The balance concept (1) relates nutrients to each other, hence capturing nutrient interactions, (2) avoids the need for the *ceteris paribus* assumption of other nutrients being equal by adjusting any nutrient or group of nutrients to others, and (3) provides a more holistic stand-alone approach illustrated by a pan balance design (Figure 1) and synthesized by an Aitchison or Mahalanobis distance to facilitate interpreting nutrient inter-relationships as looked after in the concluding remarks of recent studies (Han et al., 2011).

### Plant nutrient signatures

At ecosystem level, the soil substrate influences the distribution of terrestrial plants while genotype adaptation and genetic manipulation greatly improved crop performance in nutritionally diverse habitats (Epstein and Bloom, 2005). Wild and domesticated fruit species acquire: allocate nutrients differently and this must impact on the way plant nutrition experiments are designed and nutrients are diagnosed and managed in terms of nutrient requirements and timeframe for observable effects of nutrient supply on ionomes.

Wild species have lesser and slower response to nutrient supply compared to domesticated species (Lafond, 2009; Hébert-Gentile et al., 2011). Despite similar fundamental physiological mechanisms involved in nutrient acquisition, wild and domesticated species differ markedly in nutrient allocation between roots, stems, and the harvested part (Chapin, 1980; Jackson and Koch, 1997). The main adaptation of wild species to infertile soils appears to be to constrain growth rate to the resources available without apparent dysfunction (Chapin, 1980). Low nutrient absorption rates allow wild species to survive in nutrient-limiting, slow ion-diffusing, and stressful environments where low phenotypic plasticity maintains high root-to-shoot ratios despite occasional nutrient flushes. Domesticated species have been selected for desirable traits under conditions of high soil fertility and thus often respond to low nutrient availability with very low concentrations and visual deficiency symptoms (Chapin, 1989). Domesticated species are most often bred for high productivity under relatively luxurious environments, where there is little selective advantage in efficient nutrient use, leading to high phenotypic plasticity (Chapin, 1980, 1989). Nutrient balances are more meaningful measures of nutrient signature in this context, because nutrient imbalance caused by shortage of certain nutrients or luxury consumption of others can be detected as large multivariate distance from a landmark composition.

As expected, the *alr*- and *ilr*-based DA showed two broad categories of ionomes, the wild and the domesticated ionomes. However, this classification could be interpreted as an effect of environmental conditions or sampling protocols rather than selection pressure, but our results did not support such hypothesis. Lowbush blueberry is a wild species growing in Spodosols. Cranberry is also grown in Spodosols and fertilized similarly to lowbush blueberry, but is much more productive due to domestication. Both species were sampled at the same developmental stage. Cranberry is being selected for commercially viable traits since 1835 and across the twentieth century (Roper and Vorsa, 1997). As a result, the cranberry showed more acquaintance with domesticated than wild species, as confirmed by the *ilr*-based DA. On the other hand, although cranberry, apple, orange, mango, guava, and kiwifruit were grown in very contrasting environments, their large ellipses overlapped and were neatly separated from cloudberry and low bush blueberry ellipses, indicating human vs. natural selection pressure, respectively.

At cultivar level, the ionomes of cultivars of orange and mango grown in Ultisols and Oxisols in the state of São Paulo were differentiated by the balance model, but the cause of these differences could not be established with the present data set. In case of high phenotypic plasticity of genotypes to nutrient supply, recent research in agronomy showed that plant ionomes can be tightly supervised by balance response models and critical hyper-ellipsoids in the Euclidean space (Hernandes et al., 2012; Parent et al., 2012a; Marchand et al., 2013).

## Conclusion

This paper presents a novel numerical solution to conduct unbiased multivariate analyses of plant ionomes. The *ilrs* are orthogonally arranged log contrasts that rectify nutrient interactions of interest. The use of *ilr* balances avoids distortion due to the important properties of compositional data such as redundancy, non-normal distribution, and scale-dependency. As shown in this paper, ignoring these properties and related spurious correlations may lead to biased multivariate analyses of plant ionomes. Our finding is fundamental to plant nutritionists, physiologists, ecologists, and agronomists who attempt to classify or diagnose the ionomes of wild and domesticated species. There is a need for paradigm shift in future research. The concept of growth-limiting nutrient concentrations, supported by the “Law of minimum” and illustrated by Liebig’s barrel, should be replaced by a concept of growth-limiting nutrient balances illustrated by a pan balance design, where groups of elements are balanced optimally in weighing pans. This robust nutrient balance concept provides a structured and holistic approach to the classification of plant ionomes. Developing other suitable nutrient balances in plant nutrition studies is challenging. Obviously, many studies conducted so far in plant ionomics should be revisited. Future ecological and agronomic applications of *ilr* compositional models appear to be numerous.

## Conflict of Interest Statement

The authors declare that the research was conducted in the absence of any commercial or financial relationships that could be construed as a potential conflict of interest.
